# Quarterly Report of the Obstetric Practice in the Philadelphia Dispensary, for the Seventh, Eighth, and Ninth Months, 1841

**Published:** 1841-10-23

**Authors:** Joseph Warrington

**Affiliations:** Accoucheur


*Quarterly Report of the Obstetric Practice in the Philadelphia Dispensary, for the Seventh, Eighth, and Ninth Months*, 1841. JOSEPH WARRINGTON, M. D., Accoucheur.

Fifteen women have been delivered of fifteen children, of whom nine were boys and six were girls.

The average duration of labour in eleven cases, was six hours; the extremes being two and twelve hours.

The average amout of time required for the spontaneous delivery of the placenta, in six cases, was nineteen minutes; the extremes being five and thirty minutes.

In four cases the placenta was retained, from incapacity of spontaneous expulsion. In two of these, however, it was merely necessary to introduce the index-finger within the os uteri, to bring down an edge of the placenta. In another the entire hand had to be passed up for that purpose, while in the fourth case, in consequence of an atonic state of the uterus, contractions were produced by the introduction of the hand, after an apparent failure to produce this effect, by the administration of two scruples of powdered ergot, in two drachms of the vinous tincture of the article.

There were two cases of uterine hæmorrhage after delivery. In one woman, (who at a previous pregnancy had placentæ præviæ, with profuse sanguine discharge during labour,) it was promptly arrested by the use of wine of ergot, and abdominal compression. In the other case, the patient was in advanced phthisis; it came on some time after she had been placed in bed. It was arrested only by the free use of of wine of ergot, with additional quantities of wine, (to sustain her in her prostrate condition,) and also by the use of firm compression of the abdomen and vulva.

The women all recovered.

The fetuses were found presenting the cephalic extremity in ten of the cases which were noted—nine of these presented in the first position.

There was also one presentation of the breech, and one of the feet.

One child was still-born, (the subject of the breech presentation)—another died near a month after delivery, apparently of meningitis. The other children were doing well at the time of our attendance.

